# Metabolomics-Enhanced Liquid Biopsy Identifies Early Heptocellular Injury in Females with MetALD

**DOI:** 10.3390/ijms27114695

**Published:** 2026-05-22

**Authors:** Anika Volkmar, Gregor Mattert, Florian Deisinger, Kornelius Schulze, Asmus Heumann, Werner Dammermann, Selina Strathmeyer, Steffen Heelemann, Thomas Kalinski, Stefan Lüth, Janine Kah

**Affiliations:** 1 Faculty of Health Sciences Brandenburg, Brandenburg Medical School Theodor Fontane, 16816 Neuruppin, Germany; anika.volkmar@mhb-fontane.de (A.V.); gregor.mattert@mhb-fontane.de (G.M.); werner.dammermann@mhb-fontane.de (W.D.); s.lueth@uk-brandenburg.de (S.L.); 2 Center for Translational Medicine, Brandenburg Medical School Theodor Fontane, 14770 Brandenburg an der Havel, Germany; 3 Department of Pathology, University Hospital Brandenburg an der Havel, 14770 Brandenburg an der Havel, Germany; florian.deisinger@uk-brandenburg.de (F.D.); t.kalinski@uk-brandenburg.de (T.K.); 4 I. Department of Medicine, University Medical Center Hamburg-Eppendorf, 20251 Hamburg, Germany; k.schulze@uke.de; 5 Department of General, Visceral and Thoracic Surgery, University Medical Center Hamburg-Eppendorf, 20251 Hamburg, Germany; a.heumann@uke.de; 6 Department of Gastroenterology, Diabetology and Hepatology, University Hospital Brandenburg, 14770 Brandenburg an der Havel, Germany; 7 Lifespin GmbH, 93053 Regensburg, Germany; selina.strathmeyer@lifespin.health (S.S.); steffen.heelemann@lifespin.health (S.H.)

**Keywords:** MetALD, female health, biomarkers, CSC marker, metabolomics, NMR, sex-specific distribution

## Abstract

Steatotic liver disease (SLD) is characterised by profound metabolic reprogramming, yet no single biomarker reliably distinguishes disease entities, stages or sex-specific risk profiles. By integrating serum metabolomic signatures as a liquid biopsy with tumour-associated CSC marker profiles in a sex-stratified analytical framework, we aimed to identify biologically meaningful differences and improve strategies for early, presymptomatic detection of SLD progression and HCC. The present study focuses on a targeted panel of 12 strongly dysregulated serum metabolites as candidate biomarkers of disease progression, quantified by NMR-based metabolomics and ELISA and complemented by CSC marker staining. We combined these NMR-based metabolomic ‘liquid biopsy’ data with circulating tumour-associated biomarkers, MELD-based risk assessment and tissue-level CSC marker expression across MetALD, MASLD, immune-mediated and cancerogenic liver disease, HCC and healthy controls. Female MetALD patients showed the second highest mortality after HCC, with lower survival than male cancer patients, despite MELD 3.0 assigning ~50% higher scores in women. MetALD mortality clustered with GP73, CD44, metabolomics and AA/3HB ratio, indicating a distinct, high-risk female phenotype. Integrating liquid-based metabolomic profiling, AA/3HB redox assessment, CSC markers and MELD 3.0 into sex-sensitive diagnostic pathways may improve early detection and risk stratification of alcohol-associated SLD, especially in women.

## 1. Introduction

Hepatocellular carcinoma (HCC) remains one of the leading causes of cancer-related mortality worldwide, with incidence rates projected to rise substantially by 2040 [1,2]. In European populations, steatotic liver disease (SLD), comprising metabolic dysfunction-associated steatotic liver disease (MASLD) and metabolic dysfunction and alcohol-associated liver disease (MetALD), constitutes a significant etiological substrate for hepatocellular carcinoma (HCC). The development of cancer occurs in the context of metabolic and/or alcohol-related steatosis, accompanied by its progressive inflammatory and fibrotic sequelae, and hepatocarcinogenesis due to SLD is increasingly surpassing viral etiologies [3]. Furthermore, epidemiological projections suggest that metabolic and alcohol-related liver disease will increasingly dominate HCC-related mortality, overtaking viral etiologies within the next decade [2,4].

Despite advances in antiviral therapies and surveillance programs, HCC is frequently diagnosed at advanced stages, when curative treatment options are limited [5]. Current screening strategies rely on imaging-based surveillance combined with serum biomarkers such as alpha-fetoprotein (AFP), elastography, and fibrosis-based risk scores, including FIB-4, APRI, and the MASLD fibrosis score [6,7,8]. While these approaches are useful for fibrosis stratification, they insufficiently capture tumour-associated metabolic reprogramming and therefore often fail to detect early malignant transformation [9,10].

Within the SLD spectrum, MASLD and MetALD share a common metabolic foundation but differ fundamentally in the degree of concomitant alcohol exposure. In accordance with current nomenclature consensus, MASLD is defined by hepatic steatosis alongside at least one cardiometabolic risk factor in the absence of significant alcohol intake, whereas MetALD denotes the coexistence of metabolic dysfunction and sustained moderate alcohol consumption—typically 140–350 g/week in women and 210–240 g/week in men [11]. This distinction carries direct clinical relevance: the synergistic interaction between metabolic dysregulation and alcohol-related hepatotoxicity in MetALD may amplify steatohepatitis, accelerate fibrogenesis, and potentiate hepatocarcinogenic risk beyond that observed in either condition alone [12,13]. This vulnerability is further compounded by behavioral trends: over recent decades, alcohol consumption among women has risen disproportionately compared to men, narrowing the traditional sex gap in alcohol use disorder [11]. Women are particularly susceptible to alcohol-related liver injury at lower cumulative doses and shorter exposure durations—the so-called “telescoping effect”—and, given the frequent co-occurrence of MASLD and alcohol-related liver disease, these trends likely contribute causally to a rising incidence of MetALD in this population [14,15,16].

Despite this well-characterized epidemiological and pathophysiological landscape, reliable biomarkers capable of stratifying disease risk across the SLD spectrum—particularly in a sex-sensitive manner—remain lacking in routine clinical practice [10,17]. This diagnostic gap represents a critical unmet need, as early identification of patients at highest risk for progressive fibrosis and hepatocarcinogenesis could directly inform surveillance strategies and therapeutic decision-making [6,8,10,18].

Emerging evidence further links metabolic reprogramming to the acquisition of cancer stem cell (CSC) characteristics [13]. Markers such as CD44, CD90, EpCAM, and CK7 have been implicated with tumour plasticity, therapy resistance, and immune evasion [19,20,21]. These stemness-associated features are increasingly recognised as key drivers of tumour progression. However, despite their biological relevance, CSC-associated pathways are rarely integrated into biomarker strategies for early disease detection or risk stratification.

Importantly, most clinical risk scores, surveillance algorithms, and validation cohorts have been derived predominantly from male populations [17]. As a consequence, sex-specific biological differences, including variations in hormonal milieu, body composition, alcohol metabolism, and immune regulation, remain insufficiently explored [17,22]. This imbalance contributes to the systematic under-recognition of progressive liver disease in females and to reduced performance of existing diagnostic and prognostic tools for over 50% of the patients [14,17,22].

Although numerous metabolomic studies have identified metabolic alterations in chronic liver disease, no single biomarker has yet demonstrated sufficient specificity to reliably distinguish between disease entities or stages. Therefore, this study investigates a targeted panel of strongly dysregulated metabolites as candidate prognostic biomarkers of disease progression. By integrating serum metabolomic signatures as a ‘liquid biopsy’ with tumour-associated CSC marker profiles in a sex-stratified analytical framework, we aim (i) to identify prognostically relevant, biologically meaningful differences, and (ii) to detect sex-based differences that may improve strategies for early, presymptomatic disease detection.

## 2. Results

**Clinical stratification of the clinical cohort to assess the cirrhotic etiologies.**

**Figure 1:**
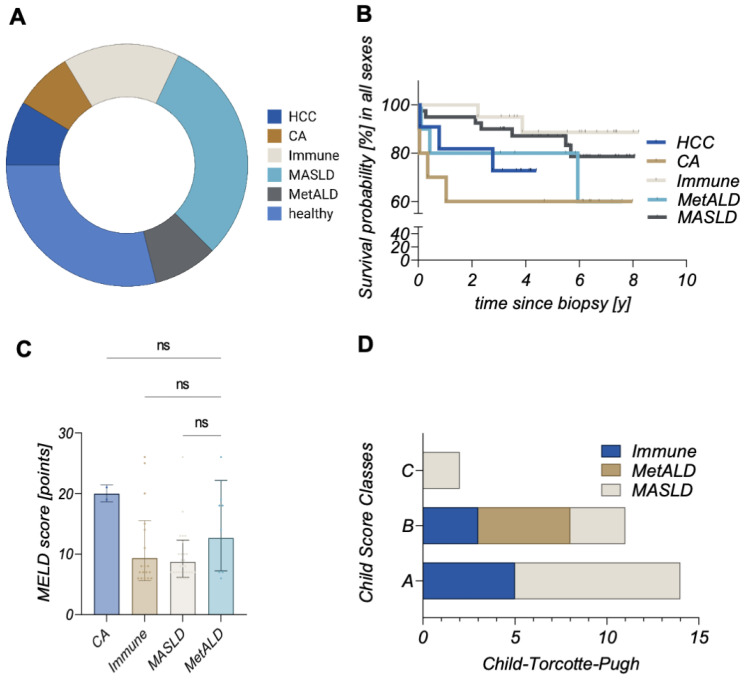
Characterization of study cohort. The composition of the cohort under study (**A**) is presented in a pie chart. The survival probability for both sexes (**B**) over an 8-year period following biopsy is broken down by entity. The clinical description of the cohort is supplemented by the MELD score (**C**) and the Child–Pugh classes (**D**). *p* values for continuous measurements were computed using ANOVA: *p* > 0.05 (ns, not significant).

The study cohort consisted of 37 healthy controls (29%), 11 patients with diagnosed hepatocellular carcinoma (HCC, 9%) and 80 patients with chronic liver disease of different etiologies, including MASLD (30%, *n* = 39), immune-mediated liver disease (Immune, 16%, *n* = 20) and MetALD (9%, *n* = 11), cancerogenic lesions (CA, 8%, *n* = 10), as illustrated in Figure 1A and defined in Table A1. All patients were classified based on clinical data (alcohol intake, infectious disease laboratory results, etc.), and imaging (LI-RADS, ultrasound), and their diagnoses were ultimately confirmed by histology (staining). Baseline demographic characteristics are summarized in Table 1. The mean age across disease groups, ranging from 46.1 years (healthy cohort) to 53.8 years in the immune-mediated cohort, 56.3 years in MetALD and 60 years in MASLD patients. The mean age of HCC patients was found to be 62.9 years, whereas the CA cohort represented the oldest group with a mean age of 67.2 years. Additionally, clinical data, including serum biomarkers, metabolic parameters, and selected tumour-associated markers, are outlined in Table 1, thus providing a comprehensive overview of the clinical and biochemical profile of the study cohort.

**The sex distribution differed between etiologies.** The healthy cohort contained the highest proportion of female individuals (64.8%), while HCC patients were predominantly male (73%), followed by MetALD with 55% males and CA displaying 60% males. In contrast, MASLD (67.5%) and immune-mediated liver disease (55%) cohorts showed a higher proportion of female patients. Kaplan–Meier analysis demonstrated etiology-dependent differences in overall survival (Figure 1B). The CA cohort exhibited the poorest survival, with approximately 60% survival at 12 months after biopsy. Patients with MetALD showed intermediate outcomes, with approximately 30% mortality within four years. In contrast, patients with MASLD and immune-mediated liver disease showed the most favorable prognosis, with immune-mediated cases reaching approximately 90% survival after eight years in this limited dataset. Liver disease severity at the time of biopsy was assessed using the MELD score (Figure 1C). The highest MELD scores were observed in the CA group (mean 20.0), followed by MetALD (mean 14.4), immune-mediated liver disease (mean 10.7), and MASLD (mean 9.3). While the number of CA cases were limited, MELD scores were significantly higher in CA compared with the other etiologies (*p* < 0.05), whereas no significant differences were observed between MASLD, MetALD, and immune-mediated liver disease. Assessment of hepatic functional reserve using the Child–Turcotte–Pugh (CTP) classification confirmed these findings (Figure 1D). The majority of MASLD and immune-mediated patients were classified as Child A or B, indicating preserved or moderately impaired liver function, whereas Child C cases were rare. In contrast, MetALD patients were predominantly classified as Child B.

Taken together, this study cohort demonstrates a clinically heterogeneous but functionally comparable data set, which allows the investigation of circulating metabolomic and tumour-associated biomarker signatures across distinct etiological phenotypes.

**Tumour-associated biomarkers differentiate hepatocellular carcinoma but do not discriminate the etiology of chronic liver disease**

**Figure 2:**
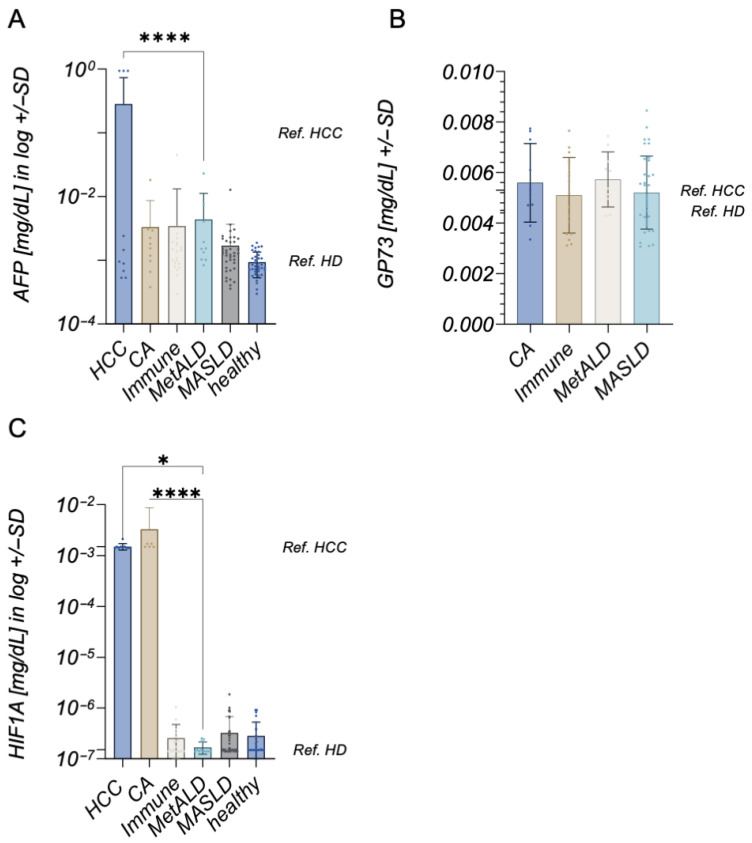
Cancer-associated marker concentrations in subgroups. Tumour-associated biomarkers categorised as AFP (**A**), GP73 (**B**), and HIF1A (**C**) [mmol/L] are listed logarithmically (**A**,**C**) and linearly (**B**) by entity. The left-hand *y*-axis shows cut-off values for HCC and healthy donors (HD), as reported in the literature. Data are presented as mean ± standard deviation (SD), with individual patient values overlaid as dots. *p* values (test by one-way ANOVA with post hoc multiple comparison) are indicated above the bars, with levels of significance denoted as * and ****.

To establish a biological framework for the subsequent metabolic analyses, we first assessed circulating biomarkers associated with tumour development, hepatocellular stress, and hypoxia-driven signaling, including alpha-fetoprotein (AFP), Golgi protein 73 (GP73), and hypoxia-inducible factor 1 alpha (HIF1A) (Figure 2). These markers are frequently discussed in the context of liver disease progression; however, they differ substantially in their diagnostic value for cirrhosis versus hepatocellular carcinoma (HCC). Importantly, while these biomarkers are established in clinical and experimental studies, their capacity to distinguish specific cirrhotic etiologies appears to be limited.

As illustrated in Figure 2A,C, HCC and healthy donor samples were displayed alongside the steatotic aetiologies encompassed within the cohort. In addition, the steatotic groups were contrasted with published reference ranges in AFP [9,23] and HIF1A [24] to provide an external benchmark for interpreting circulating biomarker concentrations.

Serum AFP levels were markedly elevated in HCC patients compared with all other groups (Figure 2A). AFP concentrations in the HCC cohort exceeded those observed in CA, Immune, MetALD, MASLD, and healthy control groups, where AFP levels remained low and largely comparable [9]. These observations are consistent with the established role of AFP as a marker predominantly associated with malignant hepatocellular transformation [9]. While AFP may increase in advanced liver injury or regenerative nodules, it generally lacks sufficient specificity and sensitivity to reliably classify SLD patients [9].

To evaluate hepatocyte injury and Golgi-associated stress responses, we next analyzed GP73 levels (Figure 2B). Unlike AFP, GP73 concentrations were relatively similar across CA, Immune, MetALD, and MASLD. While GP73 levels did not reach statistical significance across subgroups, the CA and MetALD cohorts demonstrated the highest values, exceeding those observed in immune-mediated liver disease and MASLD, in accordance with the established association of GP73 with hepatocyte injury, inflammation, and fibrotic remodelling [25]. Within this pilot cohort, GP73 levels were found to be consistently higher in advanced liver disease compared to the HCC subgroup, suggesting that GP73 primarily reflects chronic hepatocellular stress and fibrotic remodelling rather than tumour burden—supporting its proposed role as a non-specific marker of advanced liver injury across steatotic aetiologies [26], with limited ability to discriminate between individual causes of chronic liver disease.

Circulating levels of HIF1A (Figure 2C), a central regulator of cellular adaptation to hypoxia and metabolic stress [23]. HIF1A concentrations were strongly elevated in HCC patients and, to a lesser extent, in CA patients; they remained markedly lower in MetALD, MASLD, immune-mediated disease, and healthy controls. The pronounced elevation observed in HCC likely reflects hypoxia-driven signaling within the tumour microenvironment, where rapid cellular proliferation and abnormal vascularization lead to oxygen limitation and activation of hypoxia-responsive pathways. Accordingly, within this cohort, HIF1A appeared to reflect tumour-associated metabolic reprogramming rather than a general marker of cirrhosis, but in line with its role as a broadly conserved stress response rather than a cirrhosis-specific biomarker [13]. Therefore, the elevation in CA patients may be indicative of progressive tissue remodeling and compromised oxygen diffusion.

In sum, the analysis of AFP, GP73, and HIF1A predominantly captured tumour-associated processes—hepatocellular dedifferentiation and hypoxia-driven reprogramming, respectively—and thus appeared to serve primarily as indicators of HCC rather than cirrhosis in this cohort. In contrast, GP73 was more broadly associated with general hepatocyte injury and fibrotic liver remodeling but does not distinguish well between different etiologies of chronic liver disease [26]. Importantly, MetALD patients in this cohort did not display the tumour-associated biomarker profile observed in HCC, providing a rationale for incorporating NMR-based metabolic profiling to investigate whether metabolic profile might offer additional discriminatory potential.

**MetALD exhibits a distinct metabolic signature involving lipid metabolism, nitrogen detoxification, and oxidative stress**

**Figure 3:**
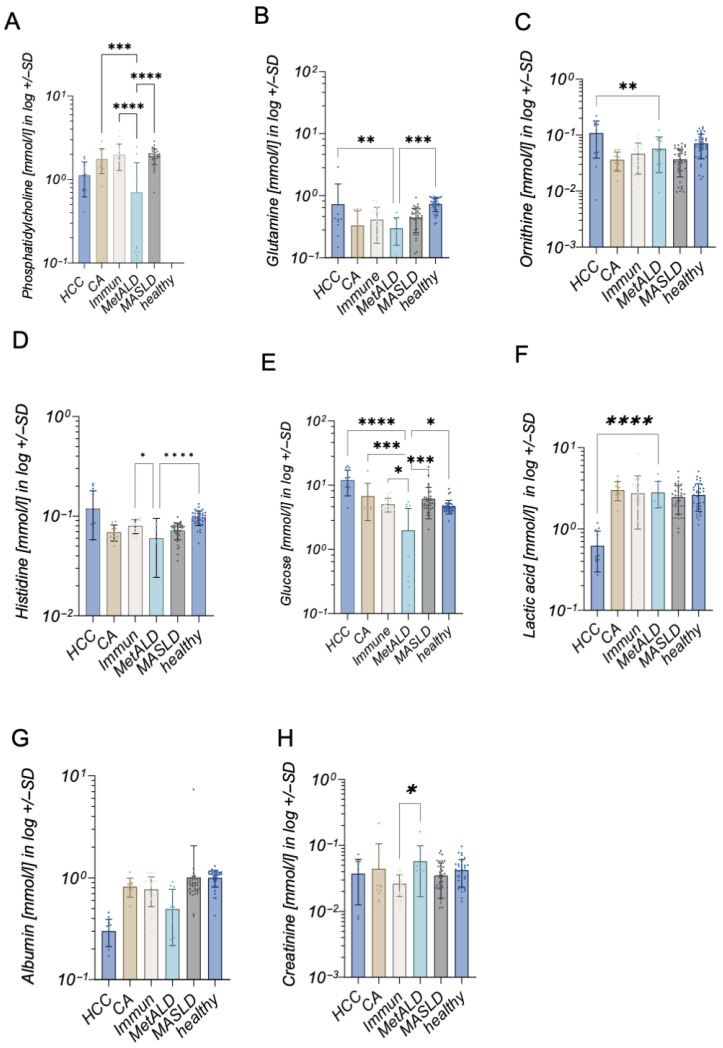
NMR-based metabolic analysis in subgroups. The bar chart depicts (**A**) phosphatidylcholine, (**B**) glutamine, (**C**) ornithine, (**D**) histidine, (**E**) glucose, (**F**) lactic acid, (**G**) albumin and (**H**) creatinine expression levels, with the highest values observed in HCC, followed by CA, MetALD and healthy controls, whereas levels are decreased in MASLD and lowest in the Immune cohort. Data are presented as mean ± standard deviation (SD), with individual patient values overlaid as dots. *p* values (test by one-way ANOVA with post hoc multiple comparison) are indicated above the bars, with levels of significance denoted as ns (*p* > 0.05), * *p* < 0.05, ** *p* ≤ 0.01, *** *p* ≤ 0.001, **** *p* ≤ 0.0001.

From an initial panel of 200 quantitative serum parameters, a targeted subset of 12 metabolites (Figure 3A–H and Figure S2) was selected based on their biological relevance to key pathophysiological domains—encompassing lipid metabolism, nitrogen detoxification, oxidative stress, energy metabolism, and hepatic synthetic function. 

To better understand the metabolic alterations associated with alcohol-associated liver disease, its cluster and progression toward HCC, these markers were systematically evaluated across different liver disease entities, thereby enabling the identification of metabolic disturbances specifically characterizing MetALD and distinguishing alcohol-induced hepatocyte injury from other chronic liver pathologies.

One of the earliest metabolic alterations observed relates to lipid metabolism, reflected by changes in phosphatidylcholine levels (Figure 3A). In the MetALD group, a significant reduction in phosphatidylcholine was observed in comparison with both CA and immune-mediated liver disease, suggesting a substantial disruption of phospholipid metabolism in alcohol-associated disease. While patients with MASLD demonstrated moderately elevated phosphatidylcholine levels, possibly indicative of metabolic syndrome-related lipid alterations, those with MetALD exhibited the lowest levels among the non-tumour liver diseases, falling even below those of patients with HCC.

Disturbances of nitrogen metabolism were captured by glutamine and ornithine (Figure 3B,C). Glutamine levels were markedly reduced in MetALD compared with all other entities, a pattern consistent with compromised ammonia buffering capacity and mitochondrial function. Ornithine concentrations in MetALD were lower than those in HCC and healthy individuals, but remained relatively higher than those observed in CA, MASLD, and immune-mediated disease. As ornithine is a central intermediate of the hepatic urea cycle, these observations suggest that nitrogen detoxification may be impaired in MetALD while potentially retaining partial functionality compared with other chronic liver diseases, where ornithine levels are even further diminished.

Histidine, a precursor for antioxidant metabolites and protective metabolites, appeared depleted in Met-ALD (Figure 3D). Histidine levels were lowest in the MetALD cohort and were significantly reduced compared with both HCC and healthy individuals. When compared with other chronic liver disease entities, histidine was also lower in MetALD than in CA, MASLD, and immune-mediated disease.

Alterations in energy metabolism were evident from changes in systemic glucose levels (Figure 3E). Glucose concentrations were lowest in MetALD patients and lower than in CA, MASLD and immune-mediated disease, consistent with ethanol-induced increases in the hepatic NADH/NAD^+^ ratio that inhibit gluconeogenic enzymes and reduce hepatic glucose production. In contrast, HCC patients displayed the highest glucose levels across all groups, in keeping with tumour-driven metabolic reprogramming and increased hepatic glucose output.

Lactate levels (Figure 3F) were comparable across all SLD subgroups, suggesting broadly preserved systemic lactate handling across non-tumor liver diseases despite ongoing metabolic stress. Lactate concentrations were lowest in HCC patients, consistent with tumour-associated metabolism in which lactate may be more actively utilized as an energy substrate within the tumour microenvironment.

Albumin levels (Figure 3G) were lowest in MetALD and HCC patients compared with healthy individuals, indicating impaired hepatocyte synthetic capacity. In MetALD, albumin concentrations were lower than in CA and immune-mediated disease but comparable to MASLD, suggesting that hepatocyte functional impairment in alcohol-associated disease may share features with metabolic liver injury.

Creatinine levels (Figure 3H) were slightly elevated in MetALD compared with CA, MASLD, and immune-mediated disease, although these differences were not statistically significant. This trend may reflect early systemic metabolic stress or subtle renal alterations that frequently accompany chronic liver disease, with creatinine indicating underlying muscle mass and protein turnover.

Methionine levels (Figure S2) were comparable across MetALD, CA, MASLD, immune-mediated disease and healthy individuals, indicating preserved hepatic methylation capacity despite the metabolic disturbances observed in MetALD.

Taken together, these observations collectively suggest a coordinated metabolic signature associated with alcohol-induced liver disease. The observed pattern confirmed that disease progression begins with impaired lipid metabolism and hepatocellular steatosis, followed by disturbances in nitrogen detoxification and oxidative stress responses. These alterations ultimately lead to impaired energy metabolism and reduced hepatic synthetic capacity.

**Altered Ketone Body Metabolism Reflects Mitochondrial Redox Imbalance in MetALD**

**Figure 4:**
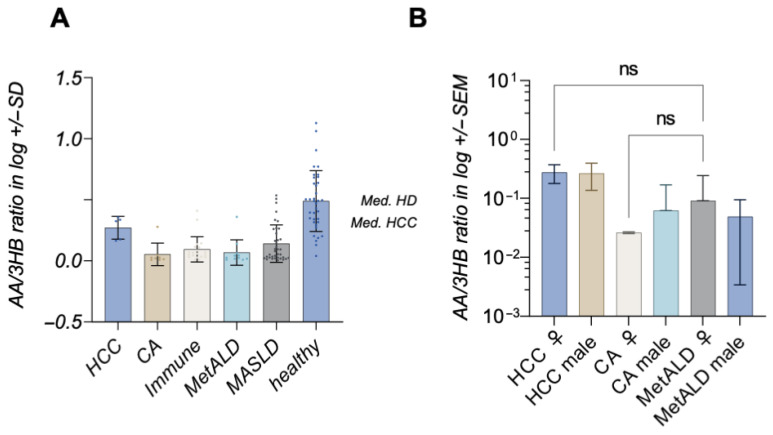
AA/3HB-ratio as a clinical feature. (**A**,**B**) Acetoacetate/3-hydroxybutyrate ratio measured by NMR in all subgroups. The ratios of females to males are additionally compared and stratified by entity. The classification of the study’s own references was performed according to the Youden index (**A**), whereas the categorization was based on the median (**B**) for each sex, as displayed on the right-hand *Y*-axis. Data are presented as mean ± standard deviation (SD), with individual patient values overlaid as dots. *p* values (test by one-way ANOVA with post hoc multiple comparison) are indicated above the bars, with levels of significance denoted as ns (*p* > 0.05).

We next examined whether the NMR-derived metabolic alterations in MetALD extend to ketone body metabolism by calculating the acetoacetate to 3-hydroxybutyrate ratio (AA/3HB), a marker of hepatic mitochondrial redox state that depends on the NADH/NAD^+^-dependent interconversion of ketone bodies [27,28]. Under physiological conditions, AA/3HB ratios in healthy individuals are typically reported around 0.5–1.0, indicating balanced ketone body metabolism [29]. When analyzing the pilot study cohort (Figure 4A), the AA/3HB ratio differed significantly across entities and was markedly reduced in MetALD compared with healthy individuals, indicating a shift toward 3-hydroxybutyrate accumulation consistent with ethanol-induced elevation of the hepatic NADH/NAD^+^ ratio and mitochondrial metabolic stress in alcohol-associated liver disease [27,30].

In contrast, HCC patients displayed comparatively higher AA/3HB ratios, approaching or exceeding those observed in healthy individuals (Figure 4A). This pattern reflects tumor-associated metabolic reprogramming and altered mitochondrial substrate utilization in HCC, potentially favouring acetoacetate accumulation or reduced conversion to 3-hydroxybutyrate. Additionally, replacement of metabolically active hepatocytes by tumor tissue may contribute to altered systemic ketone body ratios.

To assess potential sex effects on ketone body metabolism, we stratified AA/3HB ratios by biological sex (Figure 4B). This analysis was motivated by the underrepresentation of women in many alcohol-associated liver disease studies despite evidence suggesting increased susceptibility to alcohol-induced liver injury. In female MetALD, women demonstrated significantly lower median AA/3HB ratios in comparison to men, whereas median values exhibited convergence between sexes at the HCC stage. Despite a marginally more pronounced decrease in male MetALD patients, the same pattern was observed in women, indicating that the ethanol-driven mitochondrial redox shift underlying the reduced AA/3HB ratio is a general metabolic feature of MetALD rather than a sex-restricted phenomenon. The comparable alterations observed in both sexes lend support to the hypothesis that AA/3HB functions as a reliable indicator of alcohol-associated mitochondrial imbalance, thereby complementing the NMR-derived metabolomic signature.

**Tissue-level CSC marker expression correlates with systemic metabolic signatures and reveals sex-specific prediction patterns for MetALD**

To assess whether the systemic biomarker and metabolic signatures identified by liquid biopsy were reflected at the tissue level, cancer stem cell (CSC)-associated markers in liver tissue biopsies and correlated with circulating and metabolic parameters. CD44, CD90, CD133, EpCAM, and CK7 were selected as markers capturing progenitor activation, epithelial plasticity, and early tumor-associated remodeling in chronic liver injury and hepatocellular carcinogenesis. Results pertaining to representative immunohistochemical staining in CA, immune-mediated disease, and MetALD are shown in Figure S3A; quantification of positively stained tissue areas was performed using the scoring approach described in the Methods (Supplementary Figure S3B). CD44-positive areas were highest in CA tissues, whereas immune-mediated disease and MetALD samples showed lower but detectable expression (Figure S3C). In exploratory survival analysis, CD44-positive biopsies tended to have poorer surveillance outcomes than CD44-negative biopsies within this pilot study cohort (Figure S5C). CD90 expression was more pronounced in CA and MetALD than in immune-mediated disease, while CD133 levels varied across entities without clear aetiology-specific enrichment (Figure S3D,E). CK7, reflecting ductular reaction and biliary differentiation, was detectable across disease groups but most prominent in CA, and EpCAM expression was significantly higher in CA than in immune-mediated disease and MetALD, consistent with enhanced epithelial progenitor activation in advanced disease stages (Figure S3F,G). Overall, CSC-associated cellular responses were detectable across chronic liver disease entities but were most pronounced in CA, representing the end-stage disease state within this cohort.

**Figure 5:**
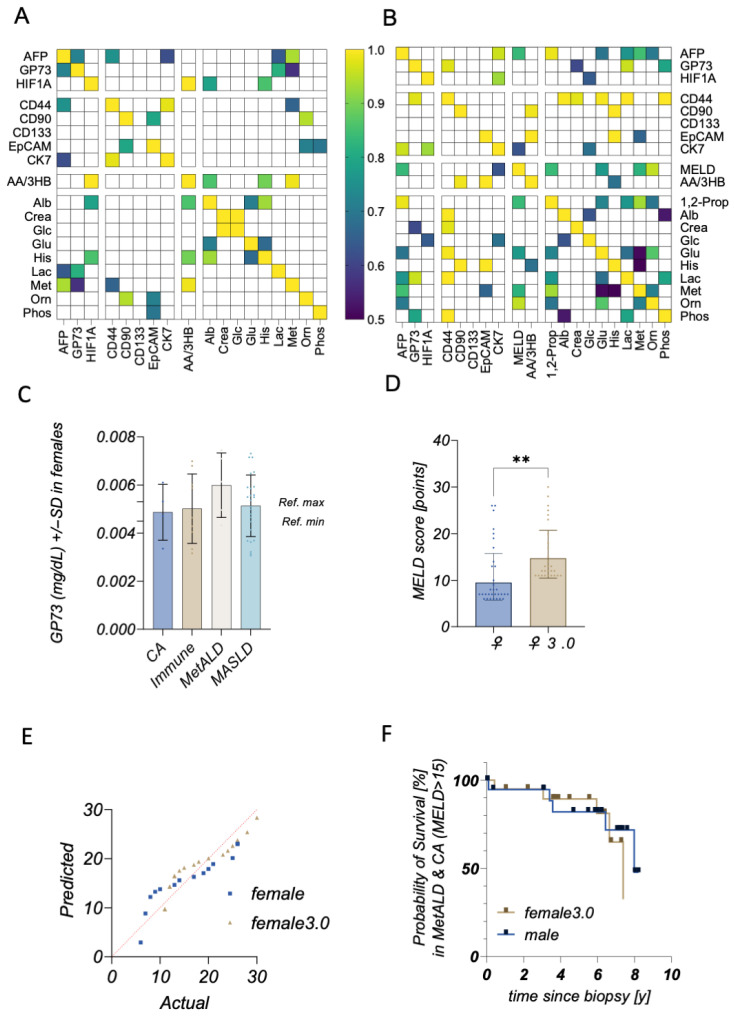
A comparison of the observed differences in sex-specific clustering in women. Tumour-associated markers, cancer stem cell (CSC) markers and metabolomics in female subjects associated with liver cell damage, including carcinogenic (**A**) and alcohol-related (**B**) damage, are shown as heatmaps. To improve visibility, the correlation coefficient r is colour-coded, ranging from 0.5 (blue) to 1 (yellow); values below 0.5 (r < 0.5) are masked (white) to highlight prominent clustering patterns. (**C**) GP73 concentrations were elevated in females and t compared with male patients. (**D**) MELD 3.0 scores were increased relative to conventional MELD scores in female (♀) patients. (**E**) Q–Q plot comparing predicted and observed values for female MELD and MELD 3.0 scores, with predicted values on the *y*-axis and observed values on the *x*-axis; data points show a slight S-shaped deviation from the reference line, indicating moderate distributional divergence. (**F**) Kaplan–Meier survival analysis for female patients with MetALD and cancerogenic liver disease and MELD score ≥ 15 over an 8-year period following biopsy, as shown in (**F**), demonstrates lower survival in females than in males. *p* values for continuous measurements were computed using ANOVA: *p* ≤ 0.01 (**).

To explore the relationship between tissue markers and systemic biomarker signatures, Pearson correlation was employed, integrating CSC markers with circulating biomarkers, metabolic parameters, and clinical indices (Figure 5A,B, Figures S4A–D and S5B). Correlation matrices for all entities are depicted in Figure S4A–D, revealing distinct, aetiology-dependent correlation patterns across the full range of coefficients. In the CA subgroup (Figure S4A), CSC markers co-clustered with markers of advanced liver dysfunction and metabolic disturbance—including albumin, creatinine, glucose and several amino acid metabolites, suggesting a close association between progenitor activation, structural liver damage and metabolic decompensation in end-stage disease.

In contrast, the MetALD subgroup (Figure S5B) displayed a distinct correlation pattern, where CSC markers were more frequently associated with metabolites related to mitochondrial redox balance and alcohol-associated metabolic remodeling, including the AA/3HB ratio, phosphatidylcholine metabolism, and amino acid metabolites such as glutamine, histidine, and ornithine. In the immune-mediated disease cohort (Figure S4C), correlations were more moderate and distributed, with CSC markers showing associations with circulating injury markers (AFP, GP73) and selected metabolic parameters. Correlations with mitochondrial redox-associated metabolites were less pronounced than in MetALD, which may reflect that progenitor activation in this entity is more closely linked to inflammatory remodelling and immune-driven hepatocellular stress. In MASLD (Figure S4D), CSC markers showed a metabolically integrated correlation pattern encompassing glucose, amino acid and phospholipid metabolism, but without prominent involvement of the AA/3HB ratio, thereby differing from the redox-driven pattern observed in MetALD.

To explore sex-dependent effects in MetALD, sex-stratified correlation analyses were performed separately in female (Figure 5A,B) and male patients (Figure S5A,B), displaying only positive correlations (*r* = 0.5–1.0) to highlight the most relevant associations. The female (Figure 5A) and male CA subgroup (Figure S5A) showed broadly comparable correlation structures. In contrast, female MetALD patients (Figure 5B) displayed a more distinguishable correlation pattern compared to their male counterparts (Figure S5B). Correlations involving the AA/3HB ratio and amino acid metabolites suggest a closer interaction between alcohol-associated mitochondrial stress and progenitor-associated tissue responses in female patients, whereas the corresponding male correlation matrices (Figure S4B) appeared less structured. This pattern suggests that metabolic–progenitor coupling may be particularly relevant for disease progression in women.

To assess the clinical relevance of these findings, disease severity and outcome were examined in the female study subgroup (Figure 5C–E). MELD 3.0 is of particular interest as it refines conventional liver disease staging by incorporating sex, serum albumin, and more granular creatinine and bilirubin weighting, thereby unmasking clinically meaningful risk gradients that conventional MELD scores may underestimate [31]. In female patients, MELD 3.0 scores were significantly higher than conventional MELD values (Figure 5D), pointing to an improved capture of disease severity in a more sex-sensitive manner. The predictive performance of MELD-based scoring in females showed good agreement between predicted and observed values in a normal QQ plot (Figure 5E). Finally, Kaplan–Meier survival analyses (Figure 5F) confirmed the validity of MELD 3.0 calculation in this cohort, with overlapping survival curves for females in MetALD and CA patients, suggesting comparable advanced disease stages and survival patterns trajectories within this cohort (Figure 5B).

Collectively, these findings are consistent with a model in which MetALD may present a metabolically defined disease phenotype, and whose integration of metabolic biomarkers with CSC-associated tissue responses may provide improved strategies for predicting disease progression, particularly in female patients with advanced disease (stages) [12,13,17].

## 3. Discussion

This pilot study provides, to our knowledge, the first integrated, sex-stratified analysis combining liquid-biopsy metabolomics, ketone-body redox profiling, conventional serum biomarkers, MELD-based risk assessment, and tissue-level CSC marker expression across a contemporary cohort of patients with MetALD, MASLD, immune-mediated liver disease, CA, HCC, and healthy controls. Within this framework, MetALD emerges as a distinct, metabolically defined disease phenotype that is insufficiently captured by conventional tumour-associated markers alone and appears to be particularly informative in female patients, in whom alcohol-related mitochondrial stress and progenitor-associated responses converge with adverse risk profiles.

Consequently, it is imperative to identify this vulnerable cohort at an early stage, while they are still in the presymptomatic stage. Based on preliminary observation, it may be reflected that in the future, metabolic analysis using a liquid biopsy should be performed clinically prior to imaging, or at the very latest, concurrently with it. Furthermore, an invasive tissue sample may be reserved as a secondary measure pending validation in larger cohorts.

This observation is clinically relevant given the rapidly changing epidemiology of liver disease. Liver cancer remains a leading cause of cancer mortality, and future projections suggest a substantial rise in HCC burden, with alcohol use and steatotic liver disease among the major drivers [1,3]. At the same time, MASLD prevalence is increasing worldwide, and MetALD has emerged as a clinically important overlap phenotype in which metabolic dysfunction and alcohol exposure synergize, amplifying steatohepatitis, fibrosis, and HCC risk [11,12,32]. This is especially relevant because MASLD-related HCC is still under-surveilled, with substantially lower screening rates than HCC or other etiologies [5].

Serum biomarker analysis confirmed that AFP and HIF1A primarily identify malignant transformation, whereas GP73 reflects general hepatocellular injury and fibrotic remodeling rather than MetALD itself. AFP behaved as expected, strongly separating HCC from non-malignant liver disease, consistent with its established role as a marker of hepatocellular dedifferentiation [9]. HIF1A likewise reflected hypoxia-driven tumor biology rather than cirrhotic etiology [23]. In contrast, GP73 was elevated across steatotic disease states, consistent with prior work showing that it tracks chronic hepatocyte stress and fibrosis rather than tumor burden alone [26]. Our GP73 ELISA values also aligned with recently discussed reference ranges and extended those data by including MetALD alongside MASLD, immune-mediated disease, CA, and HCC. Together, these findings suggest that classical tumor-associated markers are biologically informative but likely insufficient to discriminate against MetALD within this cohort [33].

The discriminatory information instead came from the metabolic layer. NMR profiling identified a MetALD-associated signature characterized by altered metabolites. This is in line with broader metabolomics literature showing that chronic liver disease is accompanied by coordinated changes in lipid metabolism, amino-acid turnover, glucose regulation, and mitochondrial pathways, and that these metabolic patterns can distinguish cirrhosis, steatohepatitis, and HCC [34,35]. In our cohort, the metabolic pattern of MetALD appeared to differ clearly from MASLD, immune-mediated disease, and HCC, which is consistent with the idea that MetALD represents a distinct pathobiological state rather than a simple overlap diagnosis.

The AA/3HB ratio added a mechanistic redox dimension to this interpretation. Because the cytosolic NADH/NAD^+^ ratio mirrors the mitochondrial pool, it serves as a functional indicator of hepatic mitochondrial redox state [27,28]. The reduced AA/3HB ratio in MetALD fits well with alcohol-driven NADH accumulation and the resulting shift toward 3-hydroxybutyrate, consistent with mitochondrial reductive stress [30,36]. By contrast, HCC showed comparatively higher ratios, likely reflecting tumor-associated metabolic reprogramming and altered mitochondrial substrate use [14]. Thus, the AA/3HB ratio complements the metabolite panel by linking the observed biochemical changes to mitochondrial dysfunction, a core feature of alcohol-associated liver injury [34].

A central translational message of this study is the explicit focus on female MetALD. Women remain underrepresented in alcohol-related liver disease research, although converging data indicate that they are biologically more vulnerable to alcohol-induced liver injury and alcohol-attributable cancer, most notably breast cancer, even at moderate consumption levels [12,16]. At the same time, hazardous drinking in middle-aged women remains common in Europe [14]. In this context, the signal observed in female MetALD in our cohort is not a marginal subgroup finding but may be directly relevant for population-level liver and cancer prevention and warrants further investigation in larger, prospective studies.

Within this pilot cohort, the data suggest that female MetALD constitutes a distinct high-risk phenotype in which metabolic dysfunction and alcohol toxicity converge more strongly than in males. This concept is mechanistically plausible: loss of oestrogen-mediated protection, increasing visceral adiposity, insulin resistance, dyslipidaemia and obesity-related inflammatory signalling are likely to amplify alcohol-induced oxidative stress, lipotoxicity and fibrogenesis in women [12,17]. In keeping with this, female MetALD patients in our cohort showed more structured and stronger correlation patterns between CSC markers and metabolic parameters than male MetALD patients, particularly involving the AA/3HB redox ratio and amino acid-related metabolites. This does not suggest an entirely different disease entity, but rather a tighter coupling between systemic metabolic stress and progenitor-associated tissue remodelling in women, whereas sex differences were less pronounced in CA, where end-stage decompensation appears to override aetiology-specific mechanisms.

The tissue-level CSC analysis supports this interpretation. Markers such as CD44, CD90, CD133, EpCAM, and CK7 are associated with progenitor activation, epithelial plasticity, migration, chemoresistance, and early hepatocarcinogenesis [20,21]. While absolute CSC-marker expression was highest in CA, the most informative observation was the correlation structure. In MetALD, CSC markers preferentially tracked metabolic stress parameters, whereas in CA, they aligned more strongly with markers of general hepatic decompensation. This suggests that, in MetALD, progenitor-associated remodeling is more tightly linked to metabolic injury biology than to terminal liver failure. In this sense, CSC markers do not function as primary MetALD classifiers, but they provide biological validation that the circulating metabolite signature reflects relevant tissue remodeling [13].

The female subgroup also highlighted the potential value of sex-dependent clinical scoring. In our cohort, MELD 3.0 was significantly higher than conventional MELD in women, which is consistent with the concept that sex-adjusted scoring better captures disease severity in female patients [31]. This is consistent with prior evidence that women are disadvantaged by creatinine-driven scoring systems because lower muscle mass leads to lower serum creatinine at comparable renal dysfunction [37]. Our findings therefore support the view that sex-dependent biomarker interpretation and sex-adapted clinical scoring merit further consideration and prospective validation.

### Limitations

This study has several strengths. It integrates clinical, serologic, metabolomic, redox, and tissue-level data within a single, well-characterised cohort. Furthermore, it employs sex-stratified analyses rather than treating sex solely as an adjustment covariate. However, it is important to acknowledge the limitations that are inherent to this approach. The number of participants in each subgroup is limited, particularly in the MetALD group and for sex-stratified analyses. All assessments are based on baseline measurements, with no longitudinal follow-up.

As a pilot study primarily aimed at establishing the methodological framework, the present findings are intended to inform hypothesis generation and should be interpreted as exploratory rather than definitive evidence, necessitating adequately powered prospective cohort studies and external validation cohorts before broader clinical conclusions can be drawn. The present study was designed to capture integrated metabolic signatures linked to early hepatocellular stress. A more detailed stratification of clinical comorbidities will therefore be addressed in future prospective studies.

Finally, due to the well-recognized discrepancy between self-reported alcohol intake and actual consumption, a clear demarcation between MASLD and MetALD cannot be ensured, which reduces the discriminatory precision between these entities and limits differentiation at the individual level. This constraint likely reflects a substantive overlap between MASLD and MetALD, as the MASLD cohort may include an unspecified proportion of patients who would formally fulfil MetALD criteria, given the open-ended questionnaire design and variability in self-reported alcohol intake. Conversely, the healthy donor group may comprise individuals in a presymptomatic disease stage who would more accurately be classified as MASLD or MetALD, which is consistent with the study’s focus on early and subclinical disease.

## 4. Materials and Methods

**Patient selection**. This retrospective cohort study included adult patients (≥18 years) presenting to the gastroenterology outpatient clinic with elevated liver enzymes or clinical suspicion of hepatic dysfunction in the context of HBV/HCV infection, autoimmune hepatitis (AIH), or hepatic malignancy. Inclusion criteria comprised: (i) availability of a diagnostic liver biopsy, (ii) a corresponding serum sample obtained at the time of biopsy, (iii) written informed consent for the use of biomaterials for research purposes, and (iv) sufficient residual tissue for experimental immunohistochemical analysis beyond routine diagnostic workup. Of an initial cohort of 177 patients, 80 met all inclusion criteria and were selected for further analysis. Patients were excluded in cases of incomplete clinical or histopathological documentation, insufficient tissue quantity, or absence of informed consent. The complete workflow of this designated pilot study is outlined in Figure S1. Overall survival was assessed by review of clinical records, with a pre-specified data cut-off of 5 July 2025. Patients without a documented event were censored at the date of last known follow-up. Within the female MetALD subgroup (*n* = 5), concomitant medication data were available for all patients. Three of five women were receiving diuretic therapy for arterial hypertension; one of these patients additionally presented with type 2 diabetes mellitus and was concurrently treated with statins and anticoagulants, representing a complex polypharmacy profile. The remaining two patients had no documented comorbidities or concomitant pharmacotherapy at the time of data collection.

**Preparation of blood samples and assessment**. Venous blood was collected from patients and healthy control subjects in serum Monovettes (31157 Sarstedt, Germany). The blood sample was left at room temperature for 30 min until coagulation was complete. After centrifugation at 1800 g for 10 min, the serum was aspirated. Aliquots of 500 µL serum were stored at −80 °C until further processing. The aliquots were then shipped on dry ice to lifespin GmbH (93053 Regensburg, Germany) for metabolome profiling and stored at −80 °C until further processing.

**Nuclear magnetic resonance (NMR) spectroscopy**. Serum aliquots were shipped on dry ice and analysed using a targeted 1H-NMR metabolomics platform (lifespin GmbH, Regensburg, Germany). After thawing at room temperature, 350 µL serum were mixed with 350 µL aqueous buffer (H_2_O p.A., 0.1 g/L NaN3, 0.067 mol/L Na2HPO4, 0.033 mol/L NaH_2_PO_4_, pH 7.15 ± 0.05, 5% D_2_O, 6 mM pyrazine), and 600 µL of the mixture were transferred into 5 mm NMR tubes (Bruker, Billerica, MA 01821, USA). Spectra were acquired at 310 K on an AVANCE NEO 600 MHz spectrometer (Bruker) using a NOESY presaturation pulse sequence (noesygppr1d, 16 scans, relaxation delay 10 s, acquisition time 2.75 s, 96 k data points, spectral width 30 ppm). 1H-NMR spectra were Fourier transformed, phased, and baseline-corrected in TopSpin 4.1.1 (Bruker). Metabolites and lipoproteins were automatically detected and quantified using proprietary software (Blood Profiler 1.4, Lipoprotein Profiler 1.2.3_A, lifespin GmbH), yielding 87 serum parameters (metabolites in mmol/L); values below the limit of detection were set to zero.

**Enzyme-linked Immuno-sorbent assay**. Precoated sandwich-ELISA were used to detect *α*-fetoprotein (AFP) (R&D Systems, Minneapolis, MN, USA), glycoprotein 73 (GP73) (ThermoFisher Scientific, Waltham, MA 02451, USA), and hypoxia-inducible factor-1-alpha (HIF1A) (ThermoFisher Scientific, Waltham, MA 02451, USA). Serum samples (diluted 1:3) were assayed as specified in the manufacturer’s protocols. No deviations from the kit procedures were introduced, except for the use of a different streptavidin–HRP solution (SDT GmbH, 52499 Baesweiler, Germany) (diluted 1:000) for the GP73 ELISA. Plates were analysed using the Tecan Spark 10 M microplate reader (Tecan Group Ltd., 8708 Männedorf, Switzerland).

**Immunohistochemistry**. Core needle liver biopsies were immediately fixed in 10% neutral buffered formalin for 24 h, routinely processed, and embedded in paraffin. Sections of 2 µm thickness were mounted on glass slides. Immunohistochemistry was performed on selected sections to evaluate the expression of stem cell markers CD44, CD90, CD133, CK7 and EpCAM. Following the HIER-based epitope-retrieval protocol by deparaffinization at 72 °C, antigen retrieval was achieved by heating slides in acidic citrate ER1 (CD133, EpCAM, CK7 (pH 6.0)) or ER2 (EDTA-based) for CD44 at 100 °C for 10 min (CD44, EpCAM, CK7) or 20 min (CD133). After inactivating endogenous peroxidase, slides were incubated with primary antibodies (each at relevant dilutions) at room temperature. Detection of rabbit and mouse primary antibodies was performed on an automated immunostainer (BOND-III, Leica Biosystems, 69226 Nussloch, Germany) using a high-sensitivity, polymer-based HRP/DAB system (Bond Polymer Refine Detection Kit, Leica Biosystems), with diaminobenzidine as chromogen and hematoxylin counterstaining, according to the manufacturer’s instructions. All runs included appropriate positive and negative controls to ensure specificity and reproducibility of the immunostaining results.

Immunohistochemical stainings were digitally acquired as single bright-field extractions at 4-fold magnification within defined target areas (Figure S3A) using an all-in-one fluorescence microscope (BZ-X1000, Keyence, Osaka 533-8555, Japan). The numbers of positively stained cells and total cells were manually counted within each target area, and expression levels were quantified as the ratio (percentage) of positive cells relative to the total cell count per field (Figure S3B). The resulting values were recorded and processed using spreadsheet software (Excel 2010 V16.58, Microsoft Corp., Redmond, WA 98052-6399, USA).

**Statistical analysis and sample size.** Processed sample size of liquid biopsies (*n* = 177) and tissue biopsies (*n* = 40) are illustrated in a flowchart (Figure S1), with entities and compared to healthy control cohort distinguished by color. Statistical analysis and visualisation were performed using GraphPad Prism 9.5.1 (Boston, MA 02110, USA). Descriptive statistics (median [IQR]) were calculated for patient characteristics. Statistical analyses were performed using one-way ANOVA with appropriate post hoc multiple comparison testing. Statistical significance was defined as *p* < 0.05, and significance levels are reported using the following notation: ns (*p* > 0.05), * *p* < 0.05, ** *p* ≤ 0.01, *** *p* ≤ 0.001, **** *p* ≤ 0.0001. Exact *p*-values are provided in the text, figures, or tables where appropriate. Pearson’s correlation analysis was used to assess associations between two parameters, with statistical significance defined according to the same thresholds. Survival analysis employed Kaplan–Meier curves with log-rank testing.

## 5. Conclusions

In summary, the findings of the present study support a model in which MetALD represents a metabolically defined disease phenotype, with female MetALD patients constituting a particularly vulnerable subgroup that is not adequately captured by conventional tumour-associated markers such as AFP, HIF1A and GP73. While these markers provide a useful contextual framework for detecting malignancy and global liver injury, they lack sufficient discriminatory power to reliably identify MetALD. By contrast, the integration of serum metabolite profiles, AA/3HB redox status, correlations with CSC markers and MELD 3.0 offers a coherent and potentially complementary approach for sex-specific risk stratification and the earlier detection of alcohol-related hepatocellular injury. In this context, sex-specific differences in susceptibility, metabolism and tissue response need to be actively incorporated into diagnostic strategies, risk stratification and therapeutic decision-making.

It should be noted, however, that this study is exploratory in nature and the statistical analyses were therefore interpreted descriptively. No formal correction for multiple testing was performed, which limits the interpretive power of the reported associations. The metabolite signatures and sex-specific patterns identified here should therefore be viewed as hypothesis-generating rather than confirmatory. Validation in larger, prospective cohorts with appropriate corrections for multiple testing will be essential to substantiate these findings and determine their clinical translation value for presymptomatic, low surveillance use prior to invasive biopsy.

## Figures and Tables

**Table 1:** Overview—characteristics of the study cohort.

		Healthy	MASLD	MetALD	CA	Immune	HCC	*p*-Value
total number		37	39	11	10	20	11	na
mean age on date of (liquid) biopsy (min–max) (y)		46.1	60	56.3	67.2	53.8	62.8	na
mean age excitus letalis (y)		-	70.7	66	64.5	82.5	62	na
Mortality rate (per 100.000)		-	1.43	3	4	0.5	3	na
month biopsy-death		-	33.43	25.67	4	35.5	10	na
sex female/male [%female]		14/13 [64.8]	26/13 [67.5]	5/6 [45.5]	4/6 [40]	11/9 [55]	3/8 [27.3]	na
Clinical score								
Child–Pugh score ‡	A	-	9	-	-	5	-	na
	B	-	3	5	1	3	-	na
	C	-	2	-	-	-	-	na
MELD 3.0 score mean (min–max)		-	9.31 (6–26)	14.38 (6–20)	20 (19–21)	10.72 (6–20)	-	0.0168
AA/3HB ratio		0.5 [0.04–1.13]	0.14 [0.0–0.36]	0.068 [0.01–0.36]	0.053 [0.0–0.28]	0.096 [0.0–0.41]	0.27 [0.16–0.36]	<0.0001
Medication								
Antidiabetic agents (Biguanides, SGLT-2 inhib., Insulin, DDP4 inhib., GLP-1-RAs)		-	5	1	1	2	-	na
Steroids (Prednisolon)		-	3	1	0	3	-	na
anticoagulant therapy ((D)OAKs, VKAs)		-	4	0	2	2	-	na
Statine		-	7	1	1	1	-	na
ACE-inhibitors		-	13	1	2	2	-	na
antiarrhythmic drugs (ß-blockers)		-	13	1	2	4	-	na
Diuretic therapy (thiazides, loops, MRAs)		-	13	1	2	5	-	na
Cancer stem cell marker evaluation								
CSC marker		Total number mean (min–max)						
CD44		-	-	3.29 [0–7]	8.2 [1–25]	5.29 [1–16]		ns
CD90		-	-	4 [1–10]	5.7 [0–15]	2.39 [0–5]		ns
CD133		-	-	0.43 [0–2]	0.5 [0–2]	1.43 [0–5]		ns
CK7		-	-	4.83 [2–9]	12.5 [2–31]	6 [2–22]		ns
EpCAM		-	-	3.13 [8–11]	16.5 [0–58]	1.43 [0–5]		<0.01
Serum-based parameters								
Parameter		Concentration						
ELISA mean [min–max]	AFP [ng/dL]	0.93 [0.3–01.9]	1.7 [0.36–12.7]	4.43 [0.83–22.9]	3.3 [0.38–18.2]	3.4 [0.3–44.6]	28.2 [0.53–94]	<0.0001
	HIF1A [mg/dL]	2.81 × 10^−7^	3.190 × 10^−7^	0.5455	0.003443	2.54737 × 10^−7^	0.003841	<0.0001
	GP73 [mg/dL]	-	0.005 [0.003–0.008]	0.006 [0.004–0.007]	0.005 [0.003–0.008]	0.005 [0.003–0.008]	-	ns
NMR ##	1,2 Prop	0.01 [0.0–0.04]	0.0 [0–0.02]	0.01 [0–0.01]	0.02 [0–0.07]	0.0 [0–0.01]	0.05 [0.0–0.21]	ns
	Albumin	1 [0.43–1.3]	1 [0.42–7.37]	0.5 [0–0.91]	0.82 [0.52–1.14]	0.8 [0.3–1.13]	0.3 [0.17–0.46]	0.0052
	Creatinine	0.42 [0.01–0.01]	0.04 [0–0.08]	0.06 [0.02–0.16]	0.04 [0.01–0.22]	0.03 [0.01–0.04]	0.04 [0.01–0.07]	ns
	Lactic acid	2.62 [1.3–5.1]	2.64 [0.91–5.1]	2.82 [1.81–4.74]	3.02 [1.67–4.46]	2.75 [0.13–8.5]	0.62 [0.27–1.19]	<0.0001
	Glucose	4.67 [2.75–8.78]	6.12 [2.05–19.2]	1.98 [0–5.48]	6.75 [4.22–16.6]	5.03 [3.5–7.7]	12 [4.4–19.84]	<0.0001
	Glutamine	0.73 [0.35–0.97]	0.44 [0.07–0.93]	0.3 [0.11–0.53]	0.33 [0.07–0.61]	0.41 [0.45–0.81]	0.73 [0.15–2.9]	<0.0001
	Histidine	0.096 [0.052–0.133]	0.071 [0.035–0.098]	0.059 [0–0.085]	0.068 [0.049–0.089]	0.079 [0.059–0.096]	0.119 [0.057–0.212]	<0.0001
	Ornithine	0.1 [0.02–0.14]	0.04 [0.01–0.12]	0.06 [0.01–0.1]	0.04 [0.02–0.05]	0.05 [0.01–0.1]	0.11 [0.00–0.22]	<0.0001
	Methionine	0.12 [0.05–0.2]	0.23 [0.01–0.1]	0.02 [0.00–0.07]	0.02 [0.00–0.1]	0.02 [0.00–0.04]	-	ns
	Phosphatidyl-choline	-	1.91 [0.7–2.87]	0.7 [0–2.45]	1.76 [0.82–2.86]	1.96 [1.71–3.88]	1.12 [0.41–1.87]	<0.0001

Data are presented as mean ± standard deviation (SD) or median (interquartile range, IQR) for continuous variables and as number (percentage) for categorical variables. Age is given in years. MASLD, Metabolic Associated Steatotic Liver Disease; MetALD, Metabolic Dysfunction-Associated and Alcoholic Liver Disease; MELD, Model for End-Stage Liver Disease; Child–Turcotte–Pugh score (CTP) indicates liver function, with the distribution across classes A, B, and C shown as indicated; AA/3HB ratio, acetoacetate to 3-hydroxybutyrate ratio. Mortality refers to [e.g., 90-day/1-year] all-cause mortality. ‡ Only calculated for patients with cirrhotic differentiation. Metabolites ## are given in mmol/L based on NMR technology (lifespin GmbH). *p* values for continuous measurements were computed using ANOVA, ns = not significant). na = not applicable.

## Data Availability

The data supporting the findings of this study are available from the corresponding author upon reasonable request.

## Supplementary Materials

The following supporting information can be downloaded at: https://www.mdpi.com/article/10.3390/ijms27114695/s1.

## Abbreviations

The following abbreviations are used in this manuscript:

1,2-Propanediol	1,2-Propanediol
AA/3HB	Acetoacetate/3-Hydroxybutyrate
AFP	*α*-Fetoprotein
AKBR	Arterial ketone body ratio
AIH	Autoimmune hepatitis
ALB	Albumin
ALT	Alanin-Aminotransferase
ANOVA	analysis of variants
CD	Cluster of differentiation
CK	Cytokeratin
CPT	Child–Torcotte–Pugh
Crea	Creatine
CSC	Cancer stem cell
ELISA	Enzyme-linked immunosorbent assay
EPCAM	Epithelial Cell Adhesion Molecule
Glc	Glucose
Glu	Glutamine
GP73	Golgi protein 73
HBV, HCV	Hepatitis B, C virus
HCC	Hepatocellular carcinoma
Hif1*α*	Hypoxinduced factor 1 alpha
His	Histidine
Lac	Lactic acid
MASLD	Metabolic dysfunction-associated steatotic liver disease
Met	Methionine
MetALD	Metabolic dysfunction-associated alcohol-related liver disease
MELD	Model of end-stage liver disease
NMR	Nuclear magnetic resonance (spectroscopy)
Orn	Ornithine
PBC	Primary biliary cholangitis
Phos	Phosphatidylcholine
PSC	Primary sclerosing cholangitis

## Appendix A

**Table A1:** Overview of characteristics of the study cohort.

Category in Manuscript	Clinical Term	Description
HCC	Hepatocellular carcinoma	Primary malignant liver tumour arising from hepatocytes.
CA (Cancerogenic liver disease)	Lesion suspicious for hepatocellular carcinoma	Cancerogenic hepatic lesion of extrahepatic origin (e.g., metastasis (LR-M)) identified histologically by pathology.
Immune	Immune-mediated liver disease	liver injury primarily caused by an aberrant immune response against hepatic or biliary mainly autoimmune origin (AIH, PBC, PSC).
MetALD	Metabolic dysfunction-associated alcohol-related liver disease	Chronic liver disease characterized by hepatic steatosis in the presence of metabolic dysfunction and significant alcohol consumption [11].
MASLD	Metabolic dysfunction-associated steatotic liver disease	Chronic liver disease associated with metabolic dysfunction and hepatic steatosis in the absence of significant alcohol intake.
Healthy donor	Healthy donor	Absence of diagnosed or symptomatic liver disease, i.e., autoimmune liver disease, infectious liver disease, steatotic liver disease including MASLD, MetALD, ALD and HCC.
