# Supplementary material for: Metabolomics-Enhanced Liquid Biopsy Identifies Early Heptocellular Injury in Females with MetALD
Source: Int J Mol Sci. 2026 May 22;27(11):4695. doi: 10.3390/ijms27114695 (PMC13256784; doi:10.3390/ijms27114695)
Supplement: Supplementary file 1 [file ijms-27-04695-s001.zip › Supplementary Figure Lengends_new.pdf]

Figure S1

Flow chart of the study design. The distinguishing characteristics of these entities are as follows: CA is represented by red, Immune by blue, MetALD by green, MASLD by yellow and healthy control group by grey. The methodology employed is delineated on the left-hand side of the image, with the selected parameters displayed in the centre. The laboratory and immunohistochemical analysis are supplemented by (calculated) clinical scores, classes and ratios.

Figure S2

Methionine levels across study cohorts. The bar chart depicts methionine expression levels are highest in HCC, followed by CA, MetALD and healthy controls, whereas levels are decreased in MASLD and lowest in the Immune cohort.

Figure S3

Immunohistochemical staining. (**A**) Illustrate various entities were subjected to staining (vertical) at 4× magnification under bright-field microscopy, with five CSC markers (horizontal) present in each. Defined target areas (**B**) of EpCAM allow the positive cell expression to be counted in relation to the total area, yielding the ratio. Ratio of IHC shows (**C**) CD44, (**D**) CD90, (**E**) CD133, (**F**) CK7 and (**G**) EPCAM.

Figure S4

Pearson correlation heatmaps. (A) CA, (**B**) MetALD, **(C)** Immune, and (**D**) MASLD cohorts. The color scale represents the correlation coefficient *r*, from −1 (blue) to 1 (yellow), and is shared between adjacent panels. Correlated variables are ordered by tumor-associated markers, CSC markers, and clinical features.

Figure S5

Pearson correlation heatmaps. (**A**) CA and (**B**) MetALD in males and CD44-associated, sex-specific survival (**C**). The color scale represents the correlation coefficient *r*, from −1 (blue) to 1 (yellow), and is shared between adjacent panels. Correlated variables are ordered by tumor-associated biomarkers, CSC markers, and clinical features. Survival (**C**) of patients with liver cell damage (immune-mediated, alcohol-related and CA) is shown in relation to high (≥5%) CD44 expression level in both sexes.
