# Supplementary figures and images for: Metabolomics-Enhanced Liquid Biopsy Identifies Early Heptocellular Injury in Females with MetALD
Source: Int J Mol Sci. 2026 May 22;27(11):4695. doi: 10.3390/ijms27114695 (PMC13256784; doi:10.3390/ijms27114695)

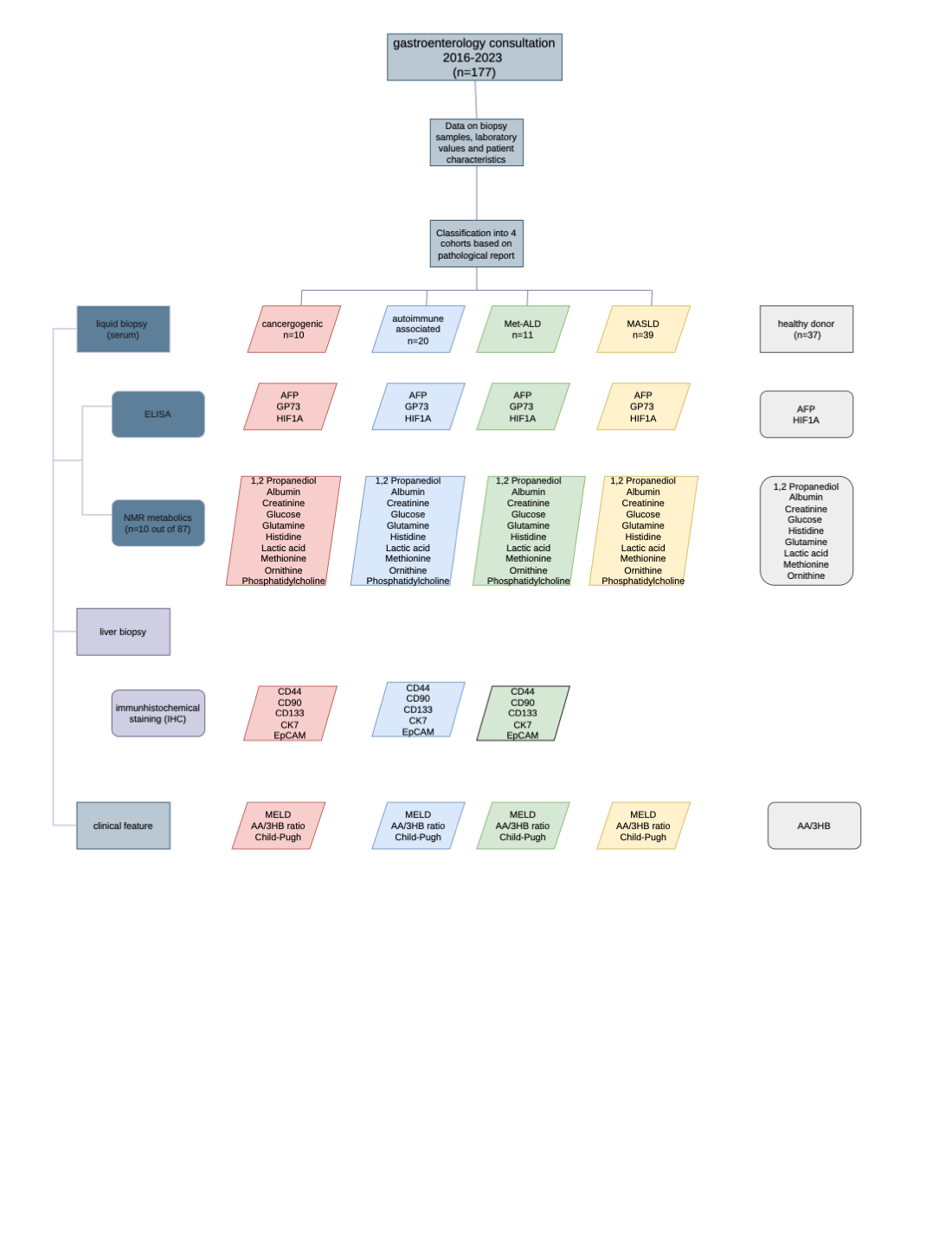

Supplement: Supplementary file 1 [file ijms-27-04695-s001.zip › Figure S1.png]

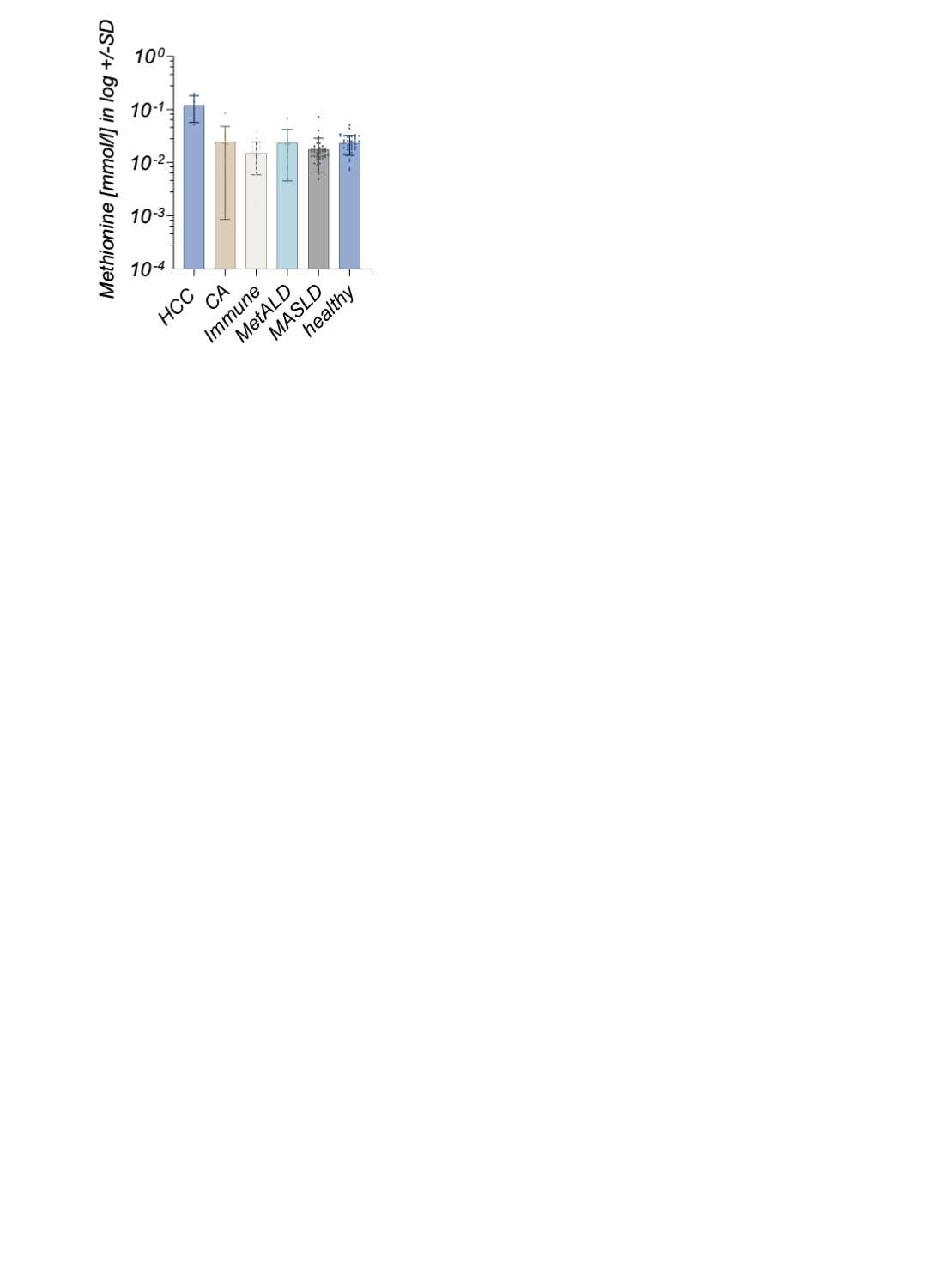

Supplement: Supplementary file 1 [file ijms-27-04695-s001.zip › Figure S2.png]

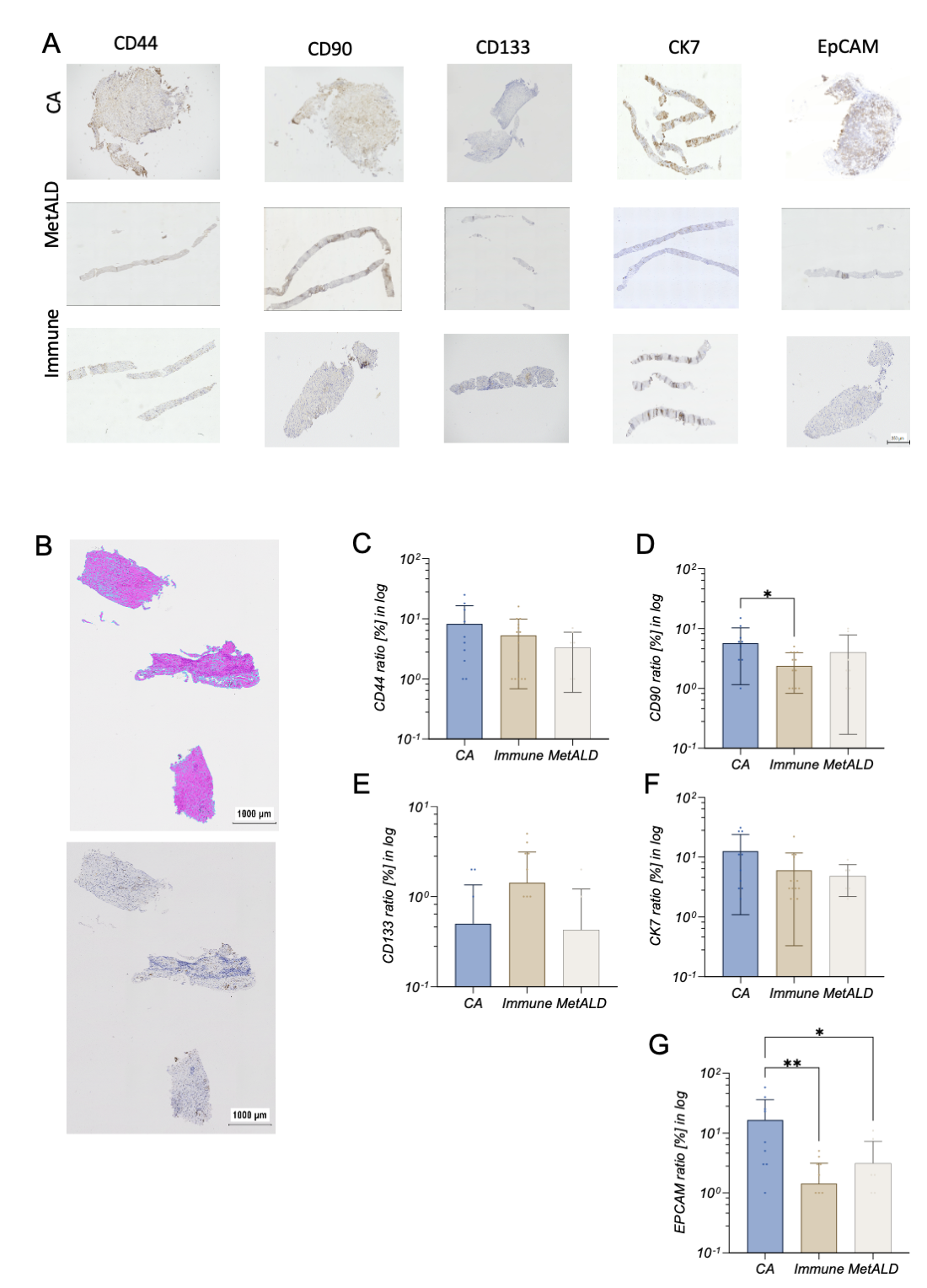

Supplement: Supplementary file 1 [file ijms-27-04695-s001.zip › Figure S3.png]

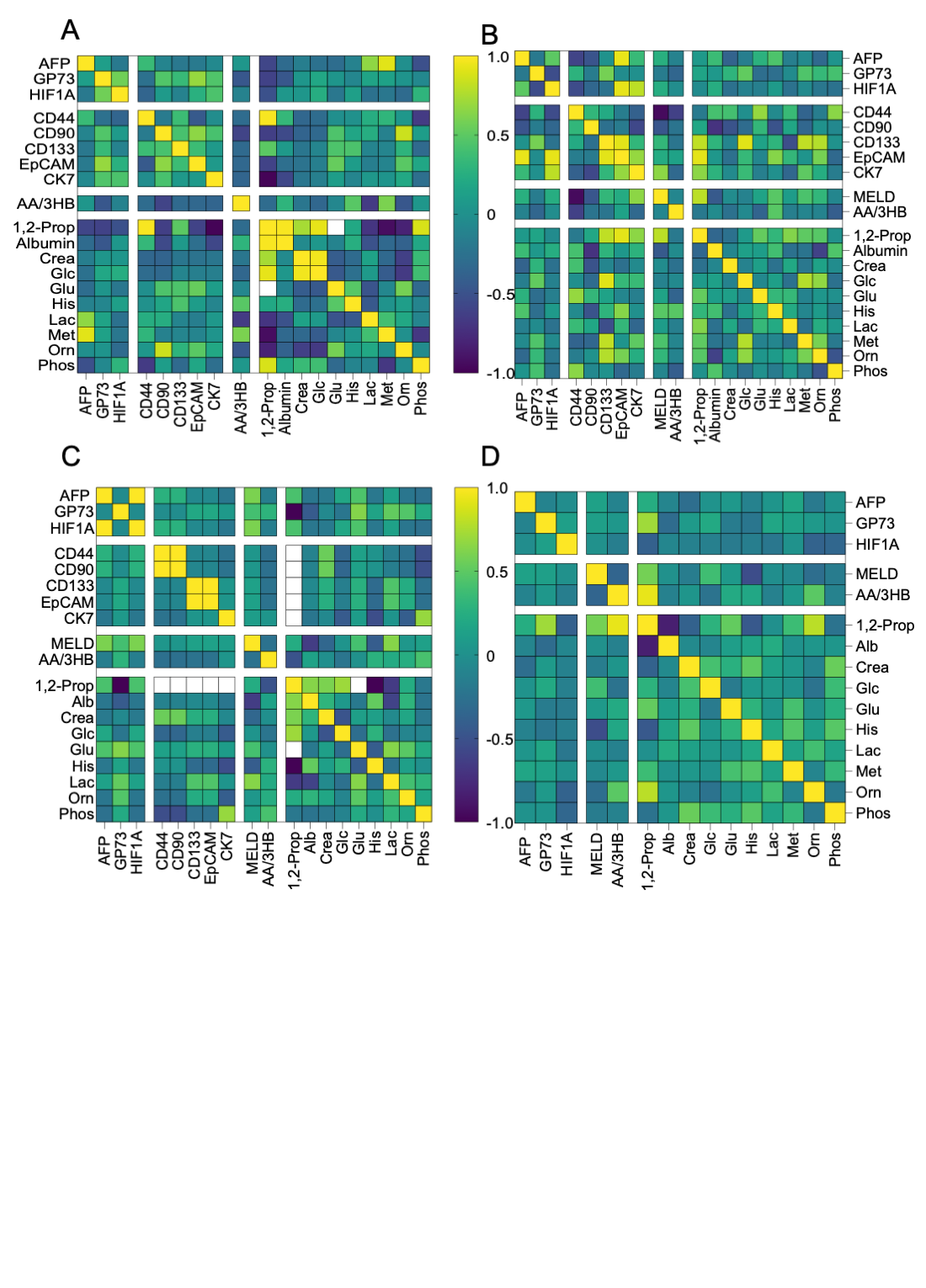

Supplement: Supplementary file 1 [file ijms-27-04695-s001.zip › Figure S4.png]

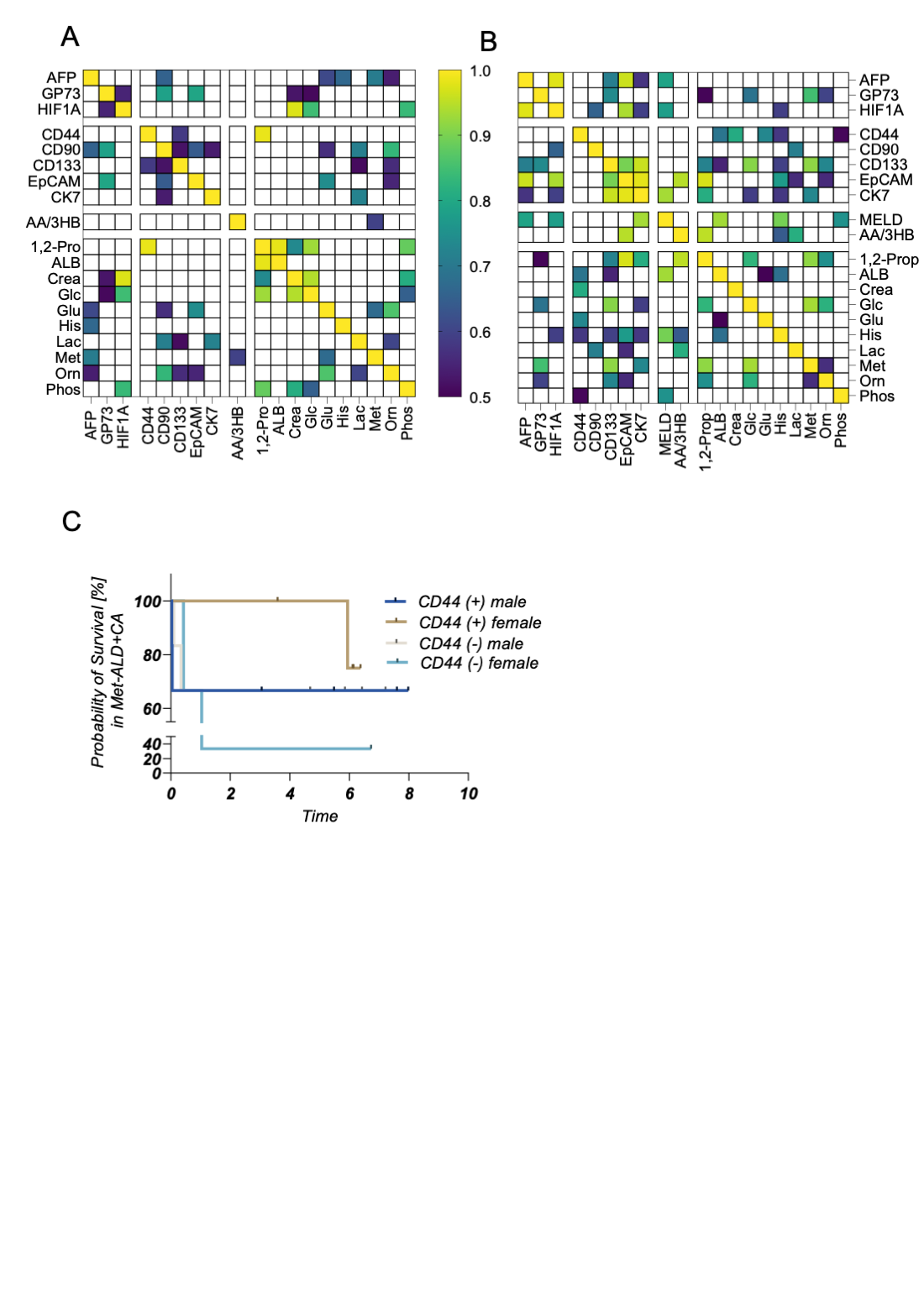

Supplement: Supplementary file 1 [file ijms-27-04695-s001.zip › Figure S5.png]
